# Clinical decisions in Orthodontics using x-ray-based images and artificial intelligence approaches: a scoping review

**DOI:** 10.1590/2177-6709.30.4.e2524219.oar

**Published:** 2026-01-09

**Authors:** Pedro Henrique José de OLIVEIRA, João Roberto GONÇALVES, Luiz Gonzaga GANDINI, Julianna de Oliveira Lima PARIZOTTO, Michelle Sousa OLIVEIRA, Renata Mayumi KATO, Karine EVANGELISTA, Lucia Helena Soares CEVIDANES, Jonas BIANCHI

**Affiliations:** 1 São Paulo State University, Dental School, Department of Morphology, Genetics, Orthodontics and Pediatric Dentistry (Araraquara/SP, Brazil).; 2 Federal University of Goiás, Dental School, Department of Orthodontics (Goiânia/GO, Brazil).; 3 University of Michigan, Dental School, Department of Orthodontics and Pediatric Dentistry (Michigan, USA).; 4 University of the Pacific, Arthur A. Dugoni Dental School, Department of Orthodontics (San Francisco, USA).

**Keywords:** Artificial intelligence, Orthodontics, X-rays, Inteligência artificial, Ortodontia, Raios X

## Abstract

**Introduction:**

Artificial intelligence (AI) in health has increased its applications over the last years. The large amount of data available due to the improvement of data storage and digital exams provided better knowledge in treatment planning and opened new possibilities of applications of AI in Orthodontics’ diagnosis and treatment planning.

**Objective:**

The aim of this scoping review was to examine when AI models enhance the clinical decision-making process in orthodontic diagnosis and treatment planning, with a focus on the utilization of X-ray-based imaging.

**Methods:**

Individual eletronic search strategies were developed and conducted on PubMed/Medline, Scopus, Web fo Science, Embase, Lilacs, and Cochrane Library (only English language articles published from January 2000 to October 20, 2021), aiming for relevant studies that met the eligibility criteria.

**Results:**

12 studies were included, categorized in 5 different groups: Orthognathic surgery, Temporomandibular Joint (TMJ) osteoarthritis, skeletal pattern, obstructive sleep apnea (OSA), and skeletal maturation/development. Most of the AI models used were Deep Learning (DL) based and the X-ray image that was most used was lateral radiographs.

**Conclusion:**

Integrating AI into clinical practice will likely continue to evolve, enhancing treatment planning in selective cases. The best applications were over TMJ osteoarthritis, skeletal maturation, and classification, OSA, and the need for orthognathic surgery.

## RESUMO

**Introdução:**

A inteligência artificial (IA) na saúde aumentou suas aplicações nos últimos anos. A grande quantidade de dados disponível, devido ao aprimoramento do armazenamento e dos exames digitais, proporcionou um melhor conhecimento no planejamento do tratamento e abriu novas possibilidades de aplicação da IA no diagnóstico e planejamento ortodôntico.

**Objetivo:**

O objetivo desta revisão de escopo foi examinar quando os modelos de IA aprimoram o processo de tomada de decisão clínica no diagnóstico e planejamento ortodôntico, com foco na utilização de imagens radiográficas.

**Métodos:**

Estratégias eletrônicas de busca individual foram desenvolvidas e realizadas nas bases PubMed/Medline, Scopus, Web of Science, Embase, Lilacs e Cochrane Library (somente artigos em inglês publicados de janeiro de 2000 a 20 de outubro de 2021), buscando estudos relevantes que atendessem aos critérios de elegibilidade.

**Resultados:**

Doze estudos foram incluídos, categorizados em cinco grupos distintos: cirurgia ortognática, osteoartrite da articulação temporomandibular (ATM), padrão esquelético, apneia obstrutiva do sono (AOS) e maturação/desenvolvimento esquelético. A maioria dos modelos de IA utilizados baseava-se em aprendizado profundo (Deep Learning - DL), sendo as radiografias laterais o tipo de imagem mais utilizado.

**Conclusão:**

A integração da IA na prática clínica provavelmente continuará a evoluir, aprimorando o planejamento do tratamento em casos seletivos. As melhores aplicações foram observadas na osteoartrite da ATM, maturação esquelética, classificação esquelética, AOS e necessidade de cirurgia ortognática.

## INTRODUCTION

Artificial intelligence (AI) is a term used to describe the ability that computational models can learn and improve their accuracy based on experience or labeled data, imitating the way that humans can learn. Its application is over streaming services, cellphones, websites, social medial etc.^1^ ^,^ ^2^

The inclusion of AI systems in the health area is an emerging topic, that has increased over the last years mainly because clinicians and researchers have more access to different exams generating a large volume of data.^1^ However, this data may be underused due to the lack of proper decision-making support systems, lack of standardization, and there is still a need for more collaborative data pooling to fuel databases.^3^ Ideally any data must follow the “4vs” (volume, velocity, variety, and veracity), which could lead to more robust data analysis.^1^

Several studies with AI have been made in different fields of dentistry,^3^ ^-^ ^5^ with different purposes, from detecting teeth and caries,^6^ ^-^ ^9^ periapical pathosis,^10^ ^-^ ^12^ and tonsils hypertrophy,^13^ ^,^ ^14^ to predict treatments, such as the need for extractions^15^ ^-^ ^18^ or orthognathic surgery.^19^ ^-^ ^25^

One subset of AI that is commonly used in the health area is convolutional (artificial) neural networks (CNN or ANN) that can be used to identify and classify images. They are called neural networks because their structure reminds the human brain networks. It can be used for raw to high-definition specific images, detection of cephalometric landmarks on lateral cephalograms, or cone-beam computed tomography (CBCTs).^26^ ^-^ ^30^ AI can also be used to assist and improve the decision-making process. It may happen because these models take large amounts of data and usually are based on expert experience or gold standard patterns.^5^ ^,^ ^18^ ^,^ ^20^ ^,^ ^31^ ^-^ ^34^

Dentistry usually provides unstructured data such as clinical data, models, photographs, and X-ray images. This type of data is difficult to analyze than structured because it is usually composed by qualitative data. However, it is’ possible to conduce researches using only unstructured data, like X-ray-based images, by using non-relational databases or by transferring unstructured data (qualitative) to structured data (quantitative).^1^ ^,^ ^35^ ^,^ ^36^

Radiographic image exams, such as CBCTs and lateral radiographs, are widely used because of the standardization and capability to provide quantitative data and images that can be extracted, segmented, and enhanced so imaging deep learning (DL) models can recognize them. In addition, the X-ray images play an essential role in diagnosis and planning.^26^ ^,^ ^27^

Still, there is a large amount of information on this field, and the aim of this scoping review was to examine when AI models enhance the clinical decision-making process in orthodontic diagnosis and treatment planning, with a focus on the utilization of X-ray-based imaging. 

## METHODS

### PROTOCOL AND REGISTRATION

The protocol of this scoping review was registered in PROSPERO under the number CRD42021259994 in the NHS (National Institute for Health Research) database at https://www.crd.york.ac.uk/prospero/. The present review follows the Preferred Reporting Items for Systematic Reviews and Meta-Analyses extension for Scoping Reviews (PRISMA-ScR) guidelines.^37^

### ELIGIBILITY CRITERIA

The PICOS plus inclusion criteria was used as inclusion criteria (Table 1). All included manuscripts were experimental or observation studies focused on AI applied to radiographic imaging exams and its application to Orthodontics diagnosis and treatment planning. Also, they should provide performance metrics or predictive outcomes that could be quantified, such as accuracy, sensibility, ROC curves, and there should be a clear mention regarding the data sets used for assessing the model. The exclusion criteria were articles that were not AI related, used more than just X-ray-based images, were not applied to diagnosis or treatment planning, were written in languages different than English, case reports, expert opinions, and letters to editors.

**Table 1:** Inclusion criteria.

PICOS	Description
Population	X-ray based studies focused on diagnostic or planning
Intervention	Use of AI for diagnostic or planning purposes
Comparator	Expert opinions, human diagnosis or gold standard
Outcome	Performance metrics (accuracy, sensibility, sensitivity...)
Study design	Observational or experimental studies
Question	How does artificial intelligence can be applied to orthodontic diagnosis and planning using x-ray based images?

### INFORMATION SOURCES AND SEARCH STRATEGY

**Figure 1:**
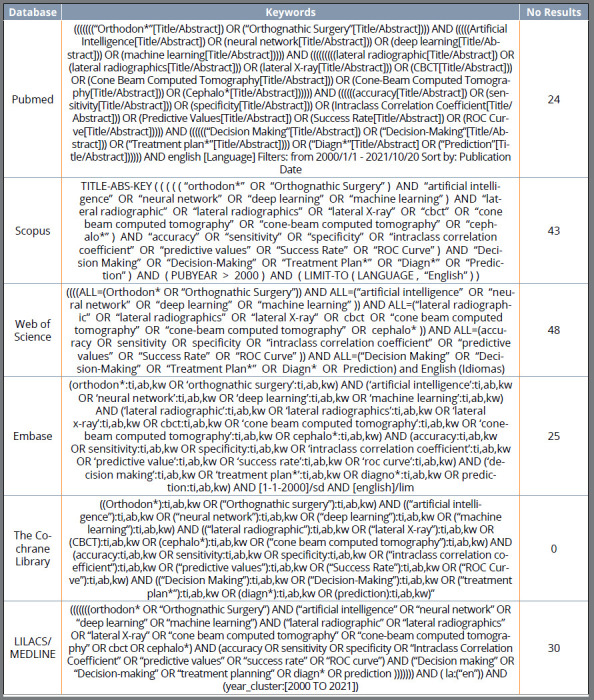
Search strategy.

Individual electronic search strategies were developed and conducted on PubMed/Medline, Scopus, Web of Science, Embase, Lilacs, and Cochrane Library published from January 2000 to October 20, 2021, and only English publish articles (Fig. 1).

### SELECTION PROCESS

The selection was completed in phases. First, titles, keywords and abstracts were reviewed by two investigators (PHJO and JOLP) separately using the inclusion and exclusion criteria to screen the articles. Duplicated articles were accessed using Mendeley (https://www.mendeley.com)^38^ and double checked using Microsoft Excel^?^. Secondly, full texts of the possible eligible studies were assessed and classified as no, maybe, or yes according to the inclusion and exclusion criteria by the two investigators. The ones marked with no for both investigators were excluded, while the ones marked as maybe were resolved through consensus to decide if they would be included or not based on the PICOS and inclusion criteria. 

### DATA CHARTING PROCESS AND DATA ITEMS

Data charting was conducted by an independent investigator (PHJO) and checked by a second (JOLP). The following data were collected from each article, as follows: author, year of publication, country, study design, sample, mean/range of age, which X-ray based exam was used, aim, algorithm architecture, performance metrics, author suggestion, recommendations and limitation, results, and conclusions. 

### STUDY RISK OF BIAS ASSESSMENT AND CRITICAL APPRAISAL OF INDIVIDUAL STUDIES

The risk of bias has not assessed, because the aim of the included manuscripts was to assess statistical machine-learning approaches based on pre-selected imaging exams, rather than the clinical aspects of the sample recruitment, or methodologies during the study’s conduction. In AI systems, the BIAS usually is resulted from the algorithm training itself, which there is no specific standard tool to measure this in scoping reviews. Meta-analysis was not conduced since the risk of bias was not assessed. Also, intervention, metrics or outcomes, and study settings were different in the selected studies.^39^

### SYNTHESIS OF RESULTS

For didactic purposes, the included studies were separated in 5 different categories, according to what was evaluated: (1) Orthognathic surgery, (2) TMJ osteoarthritis, (3) Skeletal pattern, (4) Obstructive Sleep Apnea (OSA), and (5) Skeletal maturation/development.

## RESULTS

### STUDY SELECTION

**Figure 2:**
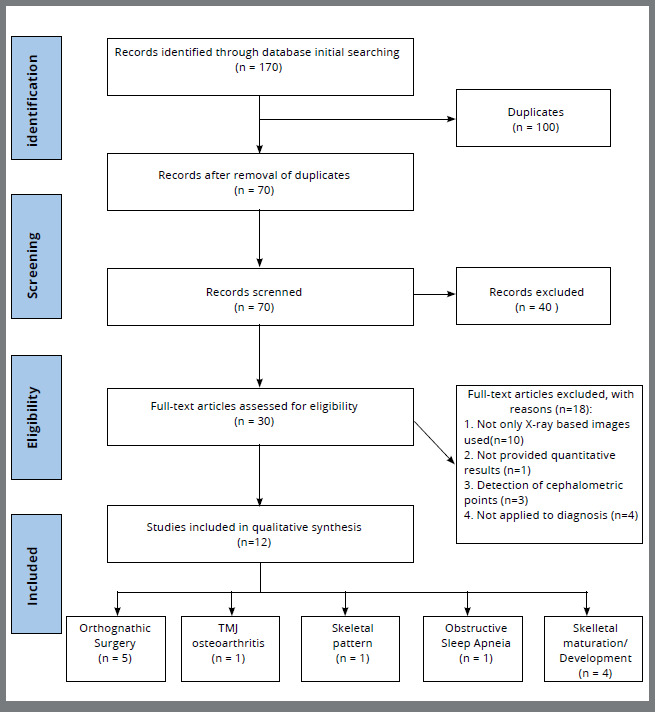
Preferred Reporting Items for Systematic Reviews and Meta-Analyses extension for Scoping Reviews (PRISMA-ScR) flow diagram of the study selection process.

A total of 170 studies were retrieved in our search following the previously defined strategy. Duplicate removal and screening were performed, and 30 studies were selected for full-text assessment. After eligibility criteria was carefully applied to the full texts, 18 studies were excluded because they were not based only on x-ray images, did not provide quantitative results, could not be applied directly to diagnosis or treatment planning, or aimed to detect cephalometric landmarks. Finally, 12 studies met the eligibility criteria and were included. The PRISMA flowchart showing the process of selection is presented in Figure 2.

### STUDY CHARACTERISTICS

**Figure 3:**
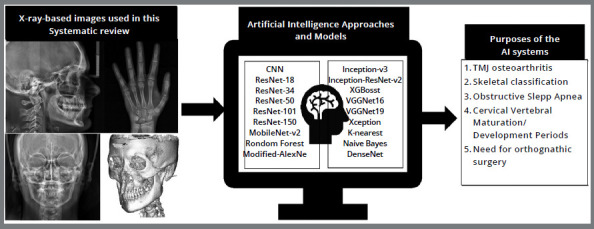
The different X-ray-based images that were used in this scoping review such as lateral radiographs (A), hand-wrist radiographs (B), Posterior radiographs (C), and CBCTs (D). Followed by different machine/deep learning methods that are present in the selected studies and the purposes of the use of AI systems in Orthodontics that are shown in this scoping review.

The studies were divided into groups according to the AI application and what was evaluated (Fig. 3).

### RESULTS OF INDIVIDUAL STUDIES AND SYNTHESIS OF RESULTS

Orthognathic surgery: Five studies were included regarding the application of AI to predict the need for orthognathic surgery or conventional Orthodontics^21^ ^-^ ^25^ (Table 2). Shin et al.^21^ demonstrated that the DL network proposed by them can standardize the decision process for orthognathic surgery and help orthodontists using posterior and lateral radiographs. Their results for accuracy, sensitivity, and specificity were 0.954, 0.844, and 0.993, respectively. Lin et al.^22^ was the only study that showed results lower than 90% for accuracy or success rate in predicting the need for orthognathic surgery. However, they evaluated that ANB angle, facial convexity angle, and the maxillary plan to Frankfurt plane angle were the main parameters to define the need for surgery. Lin et al.^23^ used four different CNN models to predict facial symmetry after orthognathic surgery using CBCT images. Xception model was the most suitable for this purpose with 90% accuracy. Kim et al.^24^ showed that ResNEt-18 and 34 showed better performance than Resnet-50 and 101 to predict the need for orthognathic surgery using lateral radiographs. The results for the four CNN models test were as follows: Area Under the Curve (AUC): 0.979 (ResNet-18), 0.974 (ResNet-34), 0.945 (ResNet-50), 0.944 (ResNet-101). Accuracy: 0.938 (ResNet-18), 0.936 (ResNet-34), 0.911 (ResNet-50), 0.913 (ResNet-101). Sensitivity: 0.882 (ResNet-18), 0.876 (ResNet-34), 0.806 (ResNet-50), 0.824 (ResNet-101). Specificity: 0.966 (ResNet-18), 0.966 (ResNet-34), 0.964 (ResNet-50), 0.958 (ResNet-101). Lee et al.^25^ showed that Deep Convolutional Neural Networks (DCNN) can be applied to predict orthognathic surgery and show which structure is the most important for diagnosis. Modified-Alexnet showed higher AUC and accuracy (0.969 and 0.919) than MobileNet (0.908 and 0.838) and ResNet-50 (0.923 and 0.838). The algorithm architecture used for all studies in this section was deep learning based.^21^ ^-^ ^25^

**Table 2:** Summary of descriptive characteristics of individual articles.

Name	Author, Year, Country	Study Design	Sample (n)	Mean/Range of Age(yr)	Imaging Analysis	Aim	Algorithm Architecture	Performance metrics	Author suggestions/recommendations	Limitations	What was evaluated	Results	Conclusions
AUTOMATED DETECTION OF TMJ OSTEOARTHRITIS BASED ON ARTIFICIAL INTELLIGENCE	Lee et al.,2020, Korea^44^	Retrospective	314 (3,514 images)	mean ± SD age, 39.5 ± 18.2 y	CBCT	to develop a diagnostic tool to detect condylar resorption from CBCT images with AI	A single-shot detector (SSD)-a deep learning framework designed for object detection	Accuracy, precision, recall, and F1 score	additional imaging modalities, such as contrast enhanced MRI and bone scintigraphy, could provide more information with higher accuracy over TMJ's inflammation.	Only 1 observer classified the TMJ images in the training data set.	TMJ osteoarthritis on CBCT on adult patients	The average accuracy, precision, recall, and F1 score over were 0.86, 0.85, 0.84, and 0.84, respectively.	TMJOA from sagittal CBCT images is possible by using a deep neural networks model.
AUTOMATED SKELETAL CLASSIFICATION WITH LATERAL CEPHALOMETRY BASED ON ARTIFICIAL INTELLIGENCE	Yu et al., 2020, Korea^43^	Retrospective	5890 patients	mean 25.4 y	Lateral radiographs	Propose a novel deep learning system that provides the fully automated, 1-step, end- to-end diagnosis for skeletal classification solely from the lateral cephalogram.	A multimodal convolutional neural network architecture with a modified DenseNet with pretrained weights for the ImageNet data set	Sensitivity, Specificity, AUC and Accuracy	To optimize diagnosis, skeletal components and severity of the discrepancy should be evaluated in future studies. Also, further research comparing this system and the original approach with landmark detection and additional studies on enhancing model performance are needed.	Data was collected from a single organization which may cause a selection bias	Three different models to evaluate sagittal and vertical skeletal pattern of patients	Sensitivity, Specificity, AUC and Accuracy for sagittal discrepancies were 93.55%, 96.77%, 0.965 to 0.991 and 95.70%, respectively. Sensitivity, Specificity, AUC and Accuracy for vertical discrepancies were 94.59%, 97.29%, 0.967 to 0.995 and 96.40%	Skeletal classification can be made with an AI-aided tool for sagittal and vertical discrepancies
MACHINE LEARNING FOR IMAGE-BASED DETECTION OF PATIENTS WITH OBSTRUCTIVE SLEEP APNEA: AN EXPLORATORY STUDY	Tsuiki et al., 2020, Japan^45^	Retrospective	1389 patients (OSA = 867; control = 522)	OSA = 49.7 ± 8.9 Control = 41.2 ± 13.0	Lateral radiographs	test the hypothesis that a machine learning model could be used to differentiate severe OSA and non-OSA by 2-dimensional images	DCNN model called Visual Geometry Group (VGG-19)	Sensitivity, Specificity, Positive and negative likelihood ratio, Positive and negative predictive values and AUC area were evaluated for the three different groups	By the combination of demographic characteristics, anthropometric features and lateral cephalometric images may provide different AI models that could contribute to detect OSA	Data includes only Asian males from a single center	If this DCNN model could be used to detect patients with OSA based on 2-D images using three different areas of interest: Original image, main region and head only	Sensitivity, Specificity, Positive and negative likelihood ratio, Positive and negative predictive values and AUC area were evaluated for the three different groups: - Full image: 0.90, 0.77, 3.88, 0.14, 0.87, 0.82 and 0.89, respectively. - Main Region: 0.84, 0.81, 4.35, 0.20, 0.88, 0.75 and 0.92, respectively. - Head only: 0.71, 0.63, 1.91, 0.46, 0.85, 0.42 and 0.70, respectively	The DCNN accurately identified individuals with severe OSA using lateral cephalometric radiographs
COMPARISON OF DEEP LEARNING MODELS FOR CERVICAL VERTEBRAL MATURATION STAGE CLASSIFICATION ON LATERAL CEPHALOMETRIC RADIOGRAPHS	Seo et al, 2021, Korea^42^	Observational	600	6-19 years	Lateral radiographs	evaluate and compare the performance of six state-of-the-art CNN-based deep learning models for CVM on lateral cephalometric radiographs, and implement visualization of CVM classification for each model using Grad-CAM technology	Different CNN models (ResNet-18, MobileNet-v2, ResNet-50, ResNet-101, Inception-v3 and Inception-ResNet-v2)	accuracy (1), recall (2), precision (3), F1-score (4), and area under the curve (AUC) values from the ROC curve	Higher quality data and development of better CNN architectures may improve the model's performance. It would be easier to evaluate if cervical vertebrae were segmented from surrounding structures	Small data	If different CNN models can distinguish the CVM classification on lateral cephalometric radiographs	Accuracy was higher than 90% for all models.	Inception-ResNet-v2 showed the best results for all metrics
DEEP CONVOLUTIONAL NEURAL NETWORKS BASED ANALYSIS OF CEPHALOMETRIC RADIOGRAPHS FOR DIFFERENTIAL DIAGNOSIS OF ORTHOGNATHIC SURGERY INDICATIONS	Lee et al., 2020, Korea^25^	Retrospective	333 individuals	23.1 ± 5.1	Lateral radiographs	Create a model for the differential diagnosis of orthognathic surgery and orthodontic treatment with a DL algorithm using cephalometric radiographs.	Three DL models were tested: Modified-Alexnet, MobileNet and Resnet50	Sensitivity, Specificity, AUC and Accuracy	Future studies can be obtained when assessed with more data. Also, should analyze the impact of better model construction and their comparison with other models	Comparison of the performance was limited due to the lack of data	Differential diagnosis for orthognathic surgery based on cephalometric radiographs using three different DL models	Modified-Alexnet: 0.852 (Sensitivity), 0.973 (Specificity), 0.969 (AUC) and 0.919 (Accuracy). MobileNet: 0.761 (Sensitivity), 0.931 (Specificity), 0.908 (AUC) and 0.838 (Accuracy). Resnet50: 0.750 (Sensitivity), 0.944 (Specificity), 0.923 (AUC) and 0.838 (Accuracy).	DCNN can be applied to predict orthognathic surgery. Also it was possible to predict the most important diagnosis structures
DEEP LEARNING BASED PREDICTION OF NECESSITY FOR ORTHOGNATHIC SURGERY OF SKELETAL MALOCCLUSION USING CEPHALOGRAM IN KOREAN INDIVIDUALS	Shin et al, 2021, Korea^21^	Observational	840 (Class II:244, Class III: 477, Assimetry: 149) (Male: 461, Female: 379)	23.2y	Posterior and lateral radiographs	Develop a deep learning network to automatically predict the need for orthodontic surgery using cephalogram.	ResNet34, with convolution blocks stacked hierarchically (CNN)	accuracy, sensitivity, and specificity.	More studies should be encouraged to achieve public confidence.	Involves only Korean patients from only one hospital and small number of cases.	Exploited a deep learning framework to automatically predict the need for orthognathic surgery based on the cephalogram of the patients	accuracy, sensitivity, and specificity were 0.954, 0.844, and 0.993, respectively	DL can standardize the decision process and help orthodontists
DETERMINATION OF GROWTH AND DEVELOPMENT PERIODS IN ORTHODONTICS WITH ARTIFICIAL NEURAL NETWORK	Kök et al., 2021, Turkey^40^	Retrospective	419 patients (CVM1:70, CVM2:70, CVM3:69, CVM4:70, CVM5:70, CVM6:70	8 to 17y	Lateral and hand-wrist radiographs	Determine the growth-development periods and gender from the cervical vertebrae using the artificial neural network (ANN).	Twenty-four ANN models were obtained and seven with success level and clinically applicable were selected	Accuracy, Sensitivity, Specificity and F1 score	Using all the information from cephalometric radiograph in addition to linear measurements can provide better results	Hand-wrist age determination is more advanced than cephalometry and cervical vertebra.	If the ANN models could determinate patient's cervical vertebral stage and gender.	The accuracy for the testing set was: 71.4% (ANN-1), 49.2% (ANN-2), 76.1% (ANN-3), 50.7% (ANN-4), 69.8% (ANN-5), 81% (ANN-6), 90.4% (ANN-7), 90.5% (ANN-Gender)	The growth-development periods and gender were determined from the cervical vertebrae by using ANN with satisfactory success
EARLY PREDICTION OF THE NEED FOR ORTHOGNATHIC SURGERY IN PATIENTS WITH REPAIRED UNILATERAL CLEFT LIP AND PALATE USING MACHINE LEARNING AND LONGITUDINAL LATERAL CEPHALOMETRIC ANALYSIS DATA	Lin et al., 2021, Korea^22^	Retrospective	56 patients	T0: 6.3y and T1: 16.7y	Lateral radiographs	Determine the cephalo- metric predictors of the future need for orthognathic surgery in patients with repaired unilateral cleft lip and palate (UCLP) using machine learning.	Boruta method to identify the most relevant features out of the cephalometric predictors at T0 and XGBoost algorithm to predict the future need for orthognathic surgery	10-fold cross-validation accuracy with F1-score	Multi-center study to increase the sample.	Small sample size.	The most important features of the cephalogram and the need for orthognathic surgery	The prediction model had 87.4% accuracy and when ANB, PP-FH, CF and FCA variables are inserted	At age of 6 years, it was possible to predict the future need for orthognathic surgery using cephalometric predictors with a good accuracy
INFLUENCE OF THE DEPTH OF THE CONVOLUTIONAL NEURAL NETWORKS ON AN ARTIFICIAL INTELLIGENCE MODEL FOR DIAGNOSIS OF ORTHOGNATHIC SURGERY	Kim et al., 2021, Korea^24^	Retrospective	960 patients (non-surgical: 640, surgical: 320)	24.6 ± 4.9	Lateral radiographs	investigate the relationship between image patterns in cephalometric radiographs and the need for orthognathic surgery, and report on a method for improving the accuracy of predictive models according to the depth of the neural network	CNN models (ResNet-18, 34, 50 and 101)	AUC, Accuracy, Sensitivity and Specificity	Multi-center data to improve the model's performance.	Single center study.	Predict the need of orthognathic surgery using cephalometric radiographs	AUC: 0.979 (ResNet-18), 0.974 (ResNet-34), 0.945 (ResNet-50), 0.944 (ResNet-101). Accuracy: 0.938 (ResNet-18), 0.936 (ResNet-34), 0.911 (ResNet-50), 0.913 (ResNet-101). Sensitivity: 0.882 (ResNet-18), 0.876 (ResNet-34), 0.806 (ResNet-50), 0.824 (ResNet-101). Specificity: 0.966 (ResNet-18), 0.966 (ResNet-34), 0.964 (ResNet-50), 0.958 (ResNet-101).	ResNet-18 and 34 had better performance than ResNet-50 and 101 models.
ON CONSTRUCTION OF TRANSFER LEARNING FOR FACIAL SYMMETRY ASSESSMENT BEFORE AND AFTER ORTHOGNATHIC SURGERY	Lin et al., 2021, Taiwan^23^	Retrospective	71 patients	-	CBCT	A CNN model with a transfer learning approach for facial symmetry assessment based on 3-dimensional (3D) features to assist physicians in enhancing medical treatments.	CNN models (VGGNet16, VGGNet19, ResNet50 and Xception)	Accuracy	Larger number of data and more effective NN architectures can enhance the accuracy	The model can only be used for small data sets and can't be trained by new DL architecture.	Predict facial symmetry scores using the trained model.	The accuracy for VGG16, VGG19, ResNet50 and Xception was: 80%, 86%, 83% and 90%, respectively.	The most suitable model for the transfer learning was Xception model
PREDICTION OF HAND-WRIST MATURATION STAGES BASED ON CERVICAL VERTEBRAE IMAGES USING ARTIFICIAL INTELLIGENCE	Kim et al., 2021, Korea^31^	Retrospective	499 images from 455 patients	9.9 ± 2.6	Lateral and hand-wrist radiographs	Predict the hand-wrist maturation stages based on the CV images observed in lateral cepha- lograms, and to analyse the accuracy of the proposed algorithms.	Various combinations of regression models were used	Mean Absolute Error (MAE), round MAE, Root Mean Square of Error (RMSE) and Accuracy	Further studies with a larger sample with na even distribution of sex, chronological age and skeletal maturation. More features can also improve the prediction accuracy.	Small sample size and imbalanced data with a great number of patients whose SMI stage was 0.	Patients hand-wrist maturation based on CV images and its accuracy.	The final ensemble model consisted of eight machine learning models. The MAE, round MAE and RMSE were 0.90, 0.87 and 1.20, respectively.	Chronological age and sex increased the accuracy. CV images can be used to predict hand-wrist SMI using machine learning.
USAGE AND COMPARISON OF ARTIFICIAL INTELLIGENCE ALGORITHMS FOR DETERMINATION OF GROWTH AND DEVELOPMENT BY CERVICAL VERTEBRAE STAGES IN ORTHODONTICS	Kök et al., 2019, Turkey^41^	Retrospective	300 patients	8 to 17y	Lateral radiographs	determine cervical vertebrae stages (CVS) for growth and development periods by the frequently used seven artificial intelligence classifiers, and to compare the performance of these algorithms with each other.	Seven algorithms were selected:k-nearest neighbors (k-NN), Naive Bayes (NB), decision tree, ANN, SVM, Random forest and logistic regression	AUC, Accuracy, Precision, F1 score and Recall	There are no author suggestions or recomendations for future studies.	The author's did not mentioned any limitations over the study	Use seven different algorithms to determine cervical vertebrae stages from cephalometric radiograph and compare their performance	Decision tree had the highest accuracy for CVS1 to CVS4 (97.1%, 90.5%, 73.2%, 58.5%, respectively) and kNN for CVS5 (60.9%) and CVS6 (78.7%). ANN had the second-highest accuracy, except for CVS5.	ANN is the preferred method for determining CVS because it was the most stable algorithm

Skeletal maturation/development: Four studies regarding the application of AI to assess development or skeletal maturation^31^ ^,^ ^40^ ^-^ ^42^ (Table 2). Kim et al.^31^ showed that cervical vertebrae (CV) images can be used to predict hand-wrist SMI by presenting a model consisted of eight ML models. Also, age and sex can increase the accuracy. Kök et al.^40^ concluded that growth development periods and gender can be predicted using ANN. The higher accuracy (0.942) was present by ANN-7 (composed by all 32 linear measurements) followed by ANN-Gender (ANN7+gender). Kök et al.^41^ results showed that ANN was the most stable algorithm to predict CV stages (CVS1-CVS6) with accuracy results of 93%, 89.7%, 68.8%, 55.6%, 47.4% and 78%, respectively. All studies used lateral radiographs and more than one DL model. However, only Seo et al.^42^ did not use hand-wrist radiographs as a comparison method. They evaluated and compared six different CNN-based DL models to classify Cervical Vertebral Maturation (CVM). All of them had accuracy higher than 90% and also AUC higher than 90% for all CVM stages. All studies showed that AI can be used to predict skeletal maturation.

Skeletal classification: One study was included regarding the application of AI to classify patients’ skeletal patterns (Table 2). Yu et al.^43^ evaluated sagittal and vertical discrepancies on lateral radiographs using CNN. It showed results higher than 90% for both skeletal classifications and presented heat maps for a better understanding of the region that influences the most to distinguish skeletal patterns.

TMJ osteoarthritis (TMJOA): One study was included regarding the application of AI to detect TMJ osteoarthritis. Lee et al.^44^ showed that sagittal CBCT images and deep learning are capable to predict TMJOA. The DL used was a single-shot detector. The authors evaluated accuracy, precision, recall, and F1 score. The results were 0.86, 0.85, 0.84, and 0.84, respectively. The authors suggested that additional imaging modalities should improve the performance of the DL and provide more information on the TMJ inflammation (Table 2).

Obstructive Sleep Apnea (OSA): One study was included regarding the application of AI to detect OSA. Tsuiki et al.^45^ showed that DCNN model (VGG-19) can accurately identify OSA using lateral radiographs (Table 2). The authors divided the sample images into three groups according to the area that was evaluated as follows: full images (original images), main region (facial profile, the upper airway, and craniofacial soft and hard tissues), and head only (occipital region). The main region had the best results for sensitivity, specificity, and AUC (0.84, 0.81, 0.92) followed by full image (0.9, 0.77, and 0.89), and head only (0.71, 0.63, and 0.7). The authors suggested that demographic characteristics and anthropometric features could improve the diagnostic OSA.

## DISCUSSION

### SUMMARY OF EVIDENCE

To our knowledge, this is the first scoping review to present X-ray imaging-based AI models applied to diagnosis and treatment planning. As a limitation, we have excluded manuscripts that used panoramic images because the studies were not focused on the Orthodontics’ diagnosis or planning. In most reviewed papers, authors divided AI applications in Orthodontics based on radiographic images into two groups: diagnosis and treatment planning. 

Diagnosis. Diagnosis is probably the most important aspect of orthodontic treatment. Assessing development and skeletal maturation is needed for patients still growing and present skeletal alterations. With that is possible to provide the best treatment for each developmental period.^46^ ^-^ ^48^ The cervical vertebrae method^49^ is commonly used to identify maturation stages because it can be assessed in lateral radiographs. However, due to its subjectivity and the chance of distortion depending on the patient positioning, clinicians need to have specific training to use this method. That is one of the reasons why hand-wrist radiographs are still the gold standard for assessing the development stage of a patient.^50^ However, AI can be used to help orthodontists to identify the CVM stages. All the studies assessed here showed that AI had good results assessing the development stage of patients using lateral radiographs when compared to gold standard or to humans’ evaluation. Furthermore, the different studies showed that ANN model had best results when compared to other AI models.^31^ ^,^ ^40^ ^-^ ^42^

Facial analysis and skeletal classification play an important role in Orthodontics’ diagnosis. All the growth deviations that the patient had will produce a skeletal alteration, and the orthodontist must classify the patient properly in all 3 planes.^51^ ^-^ ^53^ However, lateral radiographs can only classify vertical and sagittal relationships. Yu et al.^43^ aimed to used DL to assess skeletal classification and, unlikely previous studies, they did not use CNN models to perform cephalometric landmarks. The authors justified that eliminating this process would improve the classification performance. For assessing vertical relationships, it was used the Bjork’s Sum and Jarabak’s Ratio and the results showed 96.4% accuracy. For sagittal relationships, the ANB angle and Wits’ appraisal were used and a 95.7% accuracy was obtained. 

Patients with OSA usually present a retrognathic mandible and/or a lower hyoid position with causes a more crowded oropharynx. Patients with OSA can be diagnosed in lateral radiographs, mainly when it is a severe case. However, 3D images still provide better and more qualified data. Tsuiki et al.^45^ evaluated 3 types of images: full lateral radiographs images, the main airway region (including facial profile, teeth, cervical bones, maxilla, and mandible), and the occipital region. Best results were obtained for the main region, and the worst were at the occipital region. Also, their results are comparable with the manual cephalometric analysis. It shows that the use of DL models can be applied as an objective additional diagnosis method to OSA patients. 

TMJ osteoarthritis is a multi-system disease. CBCTs allow dentists to diagnose the TMJ bone by finding irregular contour, osseous defects, cortical loss or flattening of the condyles.^54^ Early diagnosis can be the best chance to minimize the damage caused by osteoarthritis, since there is no treatment available yet. Lee et al.^44^ evaluated if AI could detect TMJOA in CBCT sagittal images. The results showed that using only the CBCT sagittal images had results higher than 80% like Bianchi et al.,^55^ that used CBCT, clinical and biological data.

Treatment Planning. Once the diagnosis is placed, treatment planning is the next step in orthodontic treatment. It is important to enhance that treatment planning needs radiographic images, facial evaluation, and models to be made, mainly for borderline patients. The proper decision to treat with orthodontic treatment or orthognathic surgery usually requires more experience from the orthodontist.^51^ ^,^ ^56^ ^,^ ^57^ All studies presented here had results higher than 90% when studied adult patients that need surgical treatment. Lin et al.^22^ was the only study that used young patients with unilateral cleft lip and palate and had 87.4% accuracy and was the only one to use cephalometric measures. The other studies^21^ ^,^ ^23^ ^-^ ^25^ used DCNN to extract features and identify the structures that play an important role in the decision. The authors justify that by saying that the marking and measurement values can be a bias. This model resembles an orthodontist’s subjective or morphological analysis. Previous studies^19^ ^,^ ^58^ compared the orthodontists’ impression and cephalometric values and have concluded that there is the correlation between both analyses. 

Limitations. The main limitation of this scoping review was that most studies that apply AI to Orthodontics usually needs multi-source data, rather than x-rays-based image alone. For this reason, we retrieve only 12 manuscripts that meet our PICOs criteria. Also, we have not assessed the BIAS of each AI model due to the lack of standardized tools for this purpose. In addition to that, the lack of general applicability is a major limitation in AI models because most of the data are from the same center. We believe that with a greater number of studies conducted using AI to assist in diagnosis and planning, with more data and preferably multicenter studies, we may observe broader clinical applications and improvements in orthodontic software, thereby enhancing the proposed treatment for our patients.

## CONCLUSIONS

In fact, AI has already demonstrated its ability to add valuable information and assist in orthodontic treatment planning, diagnostic, and decision-making processes. While AI models are available to enhance the evaluation of specific issues such as TMJ osteoarthritis, skeletal maturation, skeletal classification, OSA, and the need for orthognathic surgery, it is essential to note that these applications are not universally used in orthodontic treatments. Instead, AI’s role is often context-dependent and can be most helpful in particular situations where detailed image analysis and data-driven decision support can provide significant value. Integrating AI into clinical practice will likely continue to evolve, enhancing treatment planning in selective cases.
